# Supplementary material for: Granzyme B inhibition reduces disease severity in autoimmune blistering diseases
Source: Nat Commun. 2021 Jan 12;12:302. doi: 10.1038/s41467-020-20604-3 (PMC7804321; doi:10.1038/s41467-020-20604-3)
Supplement: Supplementary file 1 — Supplementary Information [file 41467_2020_20604_MOESM1_ESM.pdf]

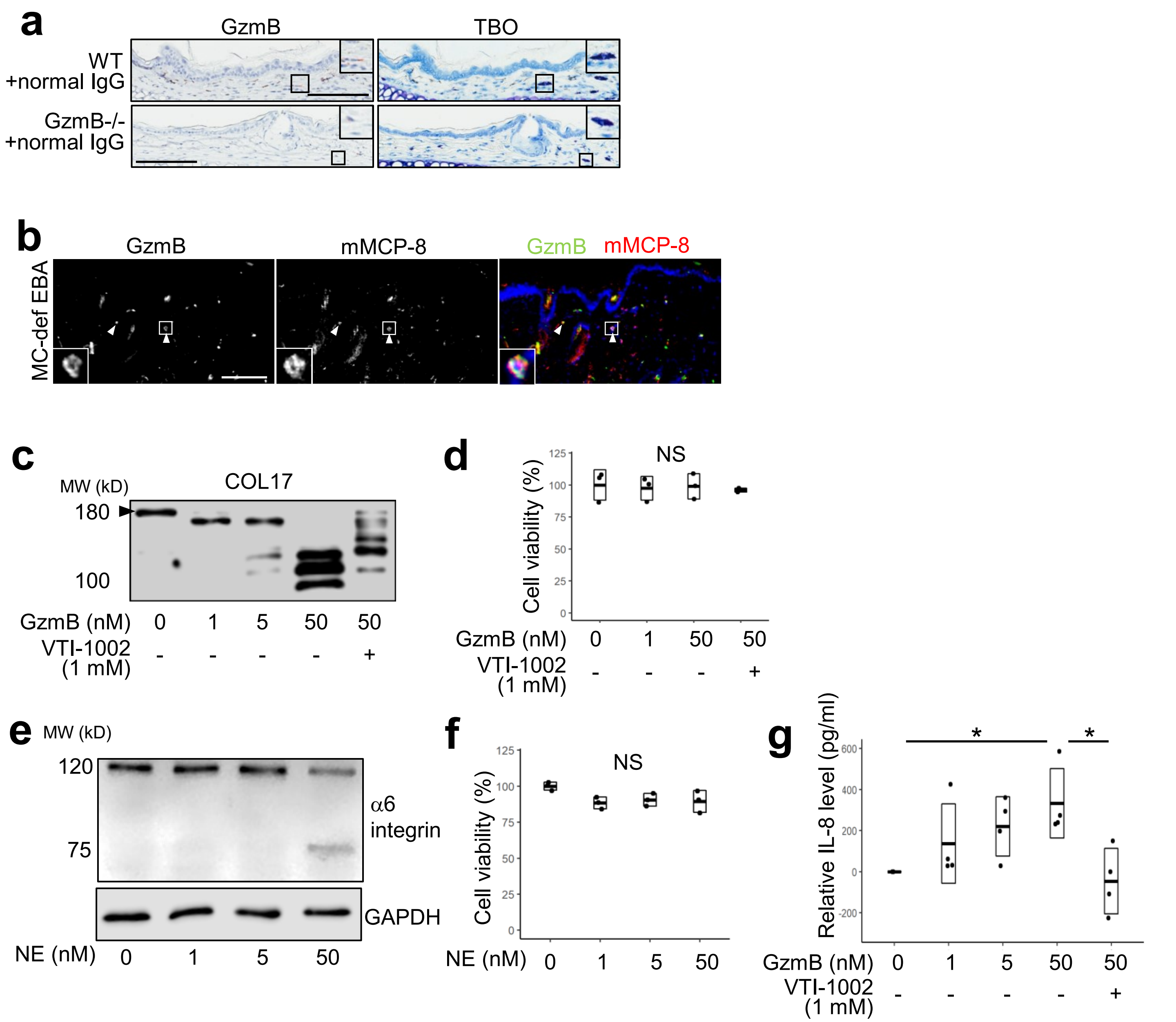

Supplementary figure 1. **a** Representative sequential GzmB immunohistochemistry (IHC) and toluidine blue O (TBO) staining images of the ears from normal IgG-injected wild-type (WT) and GzmB-/- mice at day 12. Scale bars, 100 μm. **b** Representative double staining images of lesional skin from mast cell-deficient mouse (MC-def) with systemic epidermolysis bullosa acquisita (EBA) at day 16 with antibodies against GzmB and mMCP-8 (murine basophil marker). Third columns show overlays of two stains. White arrow heads indicate cells stained with both GzmB and mMCP-8. Scale bars, 100 μm. **c** Representative immunoblotting images using antibodies against type XVII collagen (COL17) NC16a domain with recombinant human COL17, which was incubated with VTI-1002- or vehicle-pretreated recombinant human GzmB for 2 hours. Black arrowhead indicates the bands of full-length COL17. **d, f** Primary human epidermal keratinocytes (pHEKs) were incubated with VTI-1002- or vehicle-pretreated GzmB (**d**) or neutrophil elastase (NE)(**f**) for 6 hours and the cell viability was assed with MTT assay. Cell viabilities were normalized to the viability of cells incubated without GzmB, VTI-1002, or NE. *N* = 3 for each group. **e** Representative immunoblotting image using antibodies against α6 integrin with cell lysates of pHEKs, which were incubated with NE for 6 hours. GAPDH serves as loading control. **g** pHEKs were incubated with VTI-1002 or vehicle-pretreated GzmB for 16 hours and IL-8 levels in the supernatants were assessed with ELISA. Dot plot indicates individual IL-8 levels subtracted by the IL-8 level from the supernatant of the pHEKs without GzmB stimulation. *N* = 4 for each group. In all plots in (**d**), (**f**), and (**g**), dot plots indicate all individual scores and box plots indicate mean ± standard deviation. NS=not significant, *P<0.05, (One-way ANOVA with Tukey's multiple pairwise-comparisons). Images in (**a**), (**b**), (**c**), and (**e**) are representative of three independent experiments.

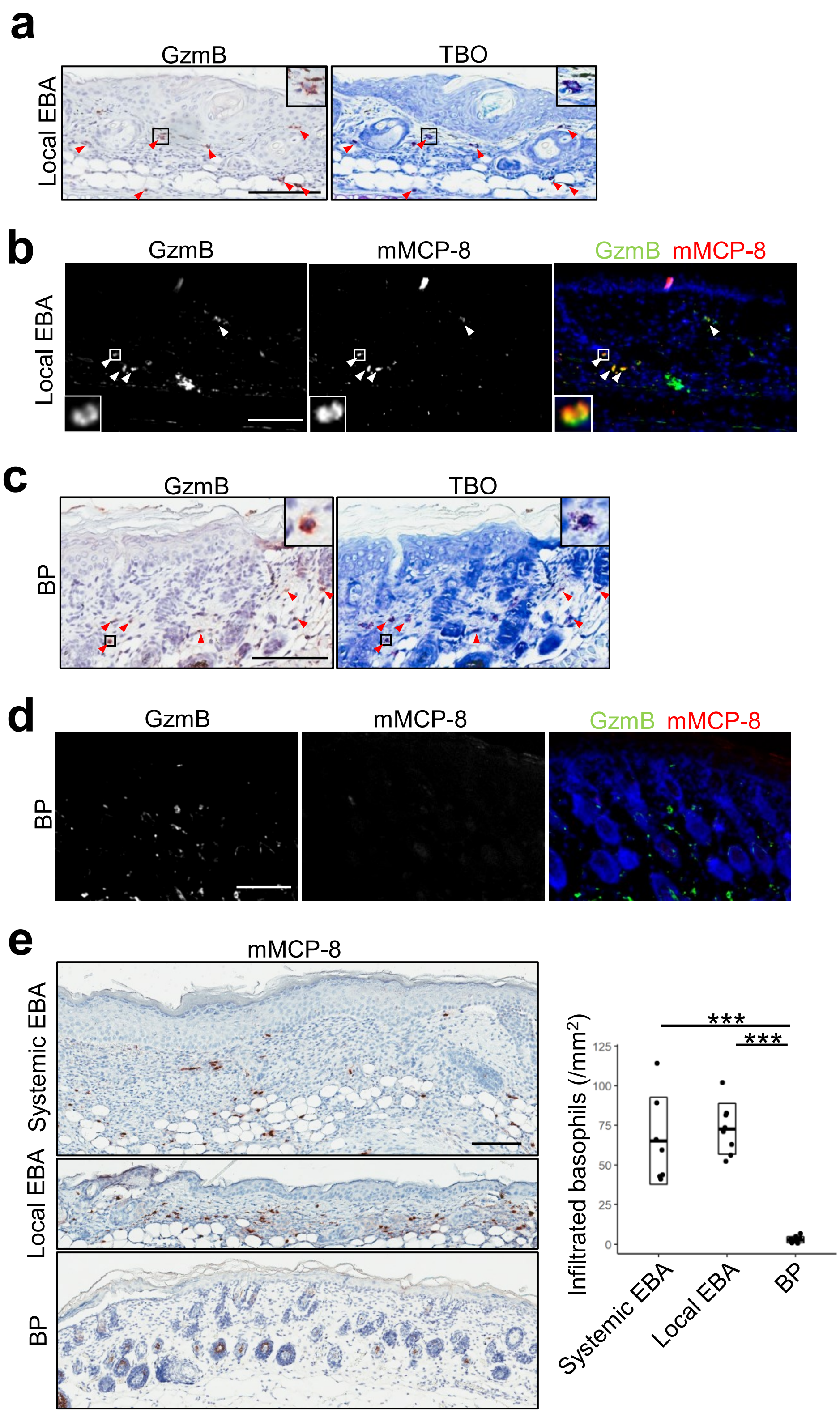

Supplementary figure 2. **a, c** Representative sequential GzmB immunohistochemistry (IHC) and toluidine blue O (TBO) staining images of the local epidermolysis bullosa acquisita (EBA) mouse ears at day 3 (**a**), and BP mice at 48 hours after the first IgG injection (**c**). Red arrowheads indicate the cells stained with both GzmB and TBO. Scale bars, 100 μm. **b, d** Representative double staining images of lesional skin from the local EBA mouse ear at day 3 (**b**), and bullous pemphigoid (BP) mice at 48 hours (**d**) with antibodies against GzmB and mMCP-8 (murine basophil marker). Third columns show overlays of two stains. White arrow heads indicate cells stained with both GzmB and mMCP-8. Scale bars, 100 μm. Images in (**a-d**) are representative of three independent experiments. **e** Representative mMCP-8 IHC images in each pemphigoid disease (PD) model. Dot plots indicate all individual scores and box plots indicate mean ± standard deviation of infiltrated basophils per unit area. *N* =7, 8, and 9 for systemic EBA, local EBA, and BP mice, respectively. ****P*<0.001 (One-way ANOVA followed by Tukey's multiple pairwise-comparisons).

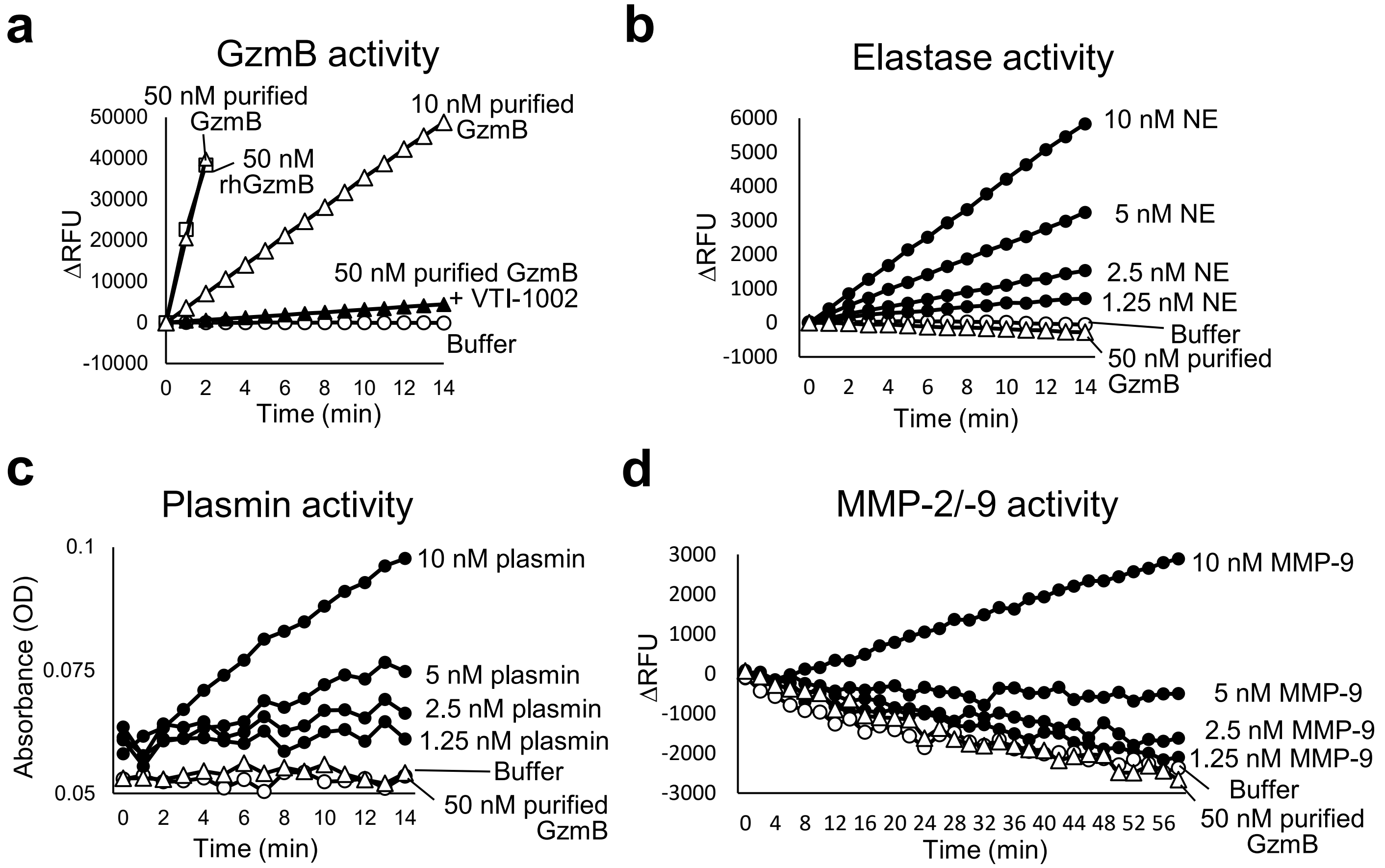

Supplementary figure 3. Protease activity in purified GzmB against GzmB (**a**), elastase (**b**), plasmin (**c**), or matrix metalloprotease (MMP)-2/-9 (d) specific substrate was assessed by kinetic protease activity assays. Relative fluorescence unit (RFU) or absorbance (optical density (OD)) was measured and ΔRFU was calculated with subtract RFU at time point 0 from RFUs at each time point. Recombinant human GzmB (rhGzmB)(**a**), elastase (**b**), plasmin (**c**), and catalytic domain of MMP-9 (**d**) were used as positive controls, respectively. (**a**) RFUs from GzmB substrate with 50 nM rhGzmB or 50 nM purified GzmB increased beyond the measuring range of machine after 3 minutes.
