# Supplementary material for: Granzyme B inhibition reduces disease severity in autoimmune blistering diseases
Source: Nat Commun. 2021 Jan 12;12:302. doi: 10.1038/s41467-020-20604-3 (PMC7804321; doi:10.1038/s41467-020-20604-3)
Supplement: Supplementary file 3 — Reporting Summary [file 41467_2020_20604_MOESM3_ESM.pdf]

nature research

Corresponding author(s): David Granville

Last updated by author(s): Oct 27, 2020

Reporting Summary

Nature Research wishes to improve the reproducibility of the work that we publish. This form provides structure for consistency and transparency in reporting. For further information on Nature Research policies, see our Editorial Policies and the Editorial Policy Checklist.

Statistics

For all statistical analyses, confirm that the following items are present in the figure legend, table legend, main text, or Methods section.

n/a	Confirmed	
☐	☒	The exact sample size (n) for each experimental group/condition, given as a discrete number and unit of measurement
☐	☒	A statement on whether measurements were taken from distinct samples or whether the same sample was measured repeatedly
☐	☒	The statistical test(s) used AND whether they are one- or two-sided<br>*Only common tests should be described solely by name; describe more complex techniques in the Methods section.*
☐	☒	A description of all covariates tested
☐	☒	A description of any assumptions or corrections, such as tests of normality and adjustment for multiple comparisons
☐	☒	A full description of the statistical parameters including central tendency (e.g. means) or other basic estimates (e.g. regression coefficient) AND variation (e.g. standard deviation) or associated estimates of uncertainty (e.g. confidence intervals)
☐	☒	For null hypothesis testing, the test statistic (e.g. F, t, r) with confidence intervals, effect sizes, degrees of freedom and P value noted<br>*Give P values as exact values whenever suitable.*
☒	☐	For Bayesian analysis, information on the choice of priors and Markov chain Monte Carlo settings
☐	☒	For hierarchical and complex designs, identification of the appropriate level for tests and full reporting of outcomes
☒	☐	Estimates of effect sizes (e.g. Cohen's d, Pearson's r), indicating how they were calculated

Our web collection on statistics for biologists contains articles on many of the points above.

Software and code

Policy information about availability of computer code

Data collection	Western blotting data were collected by Odyssey Blot Imager (LI-COR Biosciences).<br>Chromogenic staining images were collected by Aperio CS2 (Leica Biosystems).<br>Immunofluorescence images were collected by EVOS FL Imaging System (Thermo Fisher Scientific).<br>Absorbance and fluorescence were quantified by Infinite M1000 Pro plate reader (TECAN).
Data analysis	All statistics were analyzed by R (version 3.5.1, R Foundation for Statistical Computing, Vienna, Austria).<br>All images were analyzed by Image J (version 1.52p, National Institutes of Health, Bethesda, MD, USA).

For manuscripts utilizing custom algorithms or software that are central to the research but not yet described in published literature, software must be made available to editors and reviewers. We strongly encourage code deposition in a community repository (e.g. GitHub). See the Nature Research guidelines for submitting code & software for further information.

Data

Policy information about availability of data

All manuscripts must include a data availability statement. This statement should provide the following information, where applicable:

- Accession codes, unique identifiers, or web links for publicly available datasets
- A list of figures that have associated raw data
- A description of any restrictions on data availability

The data supporting the finding of this study are available within the article and from the corresponding author on reasonable request.

Field-specific reporting

Please select the one below that is the best fit for your research. If you are not sure, read the appropriate sections before making your selection.

- [x] Life sciences
- [ ] Behavioural & social sciences
- [ ] Ecological, evolutionary & environmental sciences

For a reference copy of the document with all sections, see nature.com/documents/nr-reporting-summary-flat.pdf

Life sciences study design

All studies must disclose on these points even when the disclosure is negative.

Sample size	Sample sizes were estimated based on previously published studies (Shen Exp. Mol. Med. 2018 and Hiebert Cell Death Differ. 2013) and our preliminary data obtained from pilot studies and calculated using web-based sample size/power calculator (https://www.stat.ubc.ca/~rollin/stats/ssize/).
Data exclusions	No samples or animals were excluded.
Replication	All attempts at replication were successful. At least three biologically independent samples in each group were tested in all experiments. All independent scores indicated in the figures are biologically independent.
Randomization	All samples (mice, cells, etc.) were randomly assigned to each experimental group.
Blinding	The investigators were not blinded during the group allocation, drug treatments, and data collection for the animal studies since the phenotypes were visible and available researchers were limited.<br>For the image quantification, the scanned images were quantified in a blinded fashion.

Reporting for specific materials, systems and methods

We require information from authors about some types of materials, experimental systems and methods used in many studies. Here, indicate whether each material, system or method listed is relevant to your study. If you are not sure if a list item applies to your research, read the appropriate section before selecting a response.

Materials & experimental systems

n/a	Involved in the study	
☐	☒	Antibodies
☒	☐	Eukaryotic cell lines
☒	☐	Palaeontology and archaeology
☐	☒	Animals and other organisms
☐	☒	Human research participants
☒	☐	Clinical data
☒	☐	Dual use research of concern

Methods

n/a	Involved in the study	
☒	☐	ChIP-seq
☒	☐	Flow cytometry
☒	☐	MRI-based neuroimaging

Antibodies

Antibodies used

Rabbit polyclonal antibodies against GzmB (ab4059, abcam)
Rabbit polyclonal antibodies against neutrophil elastase (ab68672, abcam)
Rabbit monoclonal antibodies against b4 integrin (EPR17517, ab182120, abcam)
Rabbit monoclonal antibodies against the 1300 – 1400 amino acid region of COL17 (EPR18614, ab184996, abcam)
Rabbit monoclonal antibodies against a6 integrin (EPR18124, ab181551, abcam)
Rat monoclonal antibody against mMCP-8 (TUG8, BioLegend)
Mouse monoclonal antibody against human ProMBP1 in basophils (J175-7D4, BioLegend)
Rabbit polyclonal antibody against NC16A domain of COL17 was a generous gift from Dr. Claus-Werner Franzke (University of Freiburg) detailed in their manuscript (Schumann et al. Am. J. Pathol. (2000))
Mouse monoclonal antibody against b-tubulin (AA2, Millipore Sigma)
Rabbit monoclonal antibody against glyceraldehyde 3-phosphate dehydrogenase (GAPDH) (14C10, #2118, Cell Signaling Technology)
Fluorescein-conjugated goat polyclonal antibody against mouse complement C3 (55500, MP Biomedicals)
Rabbit anti-human IgE heavy chain (PA5-16396, Thermo Fisher Scientific)
Alexa Fluor 488-conjugated donkey anti-rabbit-IgG (A21206, Thermo Fisher Scientific)
Alexa Fluor 594-conjugated donkey anti-rabbit-IgG (A21207, Thermo Fisher Scientific)
Alexa Fluor 488-conjugated donkey anti-rat-IgG (A21208, Thermo Fisher Scientific)
Alexa Fluor 594-conjugated donkey anti-rat-IgG (A21209, Thermo Fisher Scientific)
Biotin-conjugated goat anti-mouse IgG antibody (BA-2000, Vector Laboratories)

	Biotin-conjugated goat anti-rabbit IgG antibody (BA-1000, Vector Laboratories)<br>HRP-conjugated rabbit anti-mouse antibodies (SC-358914, Santa Cruz Biotechnology)<br>HRP-conjugated goat anti-rabbit antibodies (SC-2054, Santa Cruz Biotechnology)
Validation	All antibodies were validated by the manufacturers and documented by corresponding data sheets and/or validated in the study reported previously.<br>Rabbit polyclonal antibodies against GzmB (ab4059, abcam), mouse and human, IHC (https://www.abcam.com/granzyme-b-antibody-ab4059.html)<br>Rabbit polyclonal antibodies against neutrophil elastase (ab68672, abcam), mouse, IHC (https://www.abcam.com/neutrophil-elastase-antibody-ab68672.html)<br>Rabbit monoclonal antibodies against b4 integrin (EPR17517, ab182120, abcam), mouse, IHC and WB (https://www.abcam.com/integrin-beta-4-antibody-epr17517-ab182120.html)<br>Rabbit monoclonal antibodies against the 1300 – 1400 amino acid region of COL17 (EPR18614, ab184996, abcam), mouse and human, WB and IHC (https://www.abcam.com/collagen-xvii-antibody-epr18614-ab184996.html)<br>Rabbit monoclonal antibodies against a6 integrin (EPR18124, ab181551, abcam), mouse and human, WB and IHC (https://www.abcam.com/integrin-alpha-6-antibody-epr18124-ab181551.html)<br>Rat monoclonal antibody against mMCP-8 (TUG8, BioLegend), mouse, applied for IHC in Ugajin, J. Leukoc. Biol., (2009)<br>Mouse monoclonal antibody against human ProMBP1 in basophils (J175-7D4, BioLegend), human, IHC<br>Rabbit polyclonal antibody against NC16A domain of COL17 human, human, applied for WB in Schumann, Am. J. Pathol., (2000)<br>Mouse monoclonal antibody against b-tubulin (AA2, Millipore Sigma), mouse, WB (https://www.emdmillipore.com/CA/en/product/Anti-Tubulin-Antibody-clone-AA2,MM_NF-05-661)<br>Rabbit monoclonal antibody against GAPDH (14C10, #2118, Cell Signaling Technology), human, WB<br>Fluorescein-conjugated goat polyclonal antibody against mouse complement C3 (55500, MP Biomedicals), mouse, IF (https://www.mpbio.com/ca/0855500-fluorescein-conjugated-goat-igg-fraction-to-mouse-complement-c3)<br>Rabbit anti-human IgE heavy chain (PA5-16396, Thermo Fisher Scientific), human, IHC (https://www.thermofisher.com/antibody/product/Rabbit-anti-Human-IgE-Heavy-chain-Secondary-Antibody-Polyclonal/PA5-16396)<br>Alexa Fluor 488-conjugated donkey anti-rabbit-IgG (A21206, Thermo Fisher Scientific), IF (https://www.thermofisher.com/antibody/product/Donkey-anti-Rabbit-IgG-H-L-Highly-Cross-Adsorbed-Secondary-Antibody-Polyclonal/A-21206)<br>Alexa Fluor 594-conjugated donkey anti-rabbit-IgG (A21207, Thermo Fisher Scientific), IF (https://www.thermofisher.com/antibody/product/Donkey-anti-Rabbit-IgG-H-L-Highly-Cross-Adsorbed-Secondary-Antibody-Polyclonal/A-21207)<br>Alexa Fluor 488-conjugated donkey anti-rat-IgG (A21208, Thermo Fisher Scientific), IF (https://www.thermofisher.com/antibody/product/Donkey-anti-Rat-IgG-H-L-Highly-Cross-Adsorbed-Secondary-Antibody-Polyclonal/A-21208)<br>Alexa Fluor 594-conjugated donkey anti-rat-IgG (A21209, Thermo Fisher Scientific), IF (https://www.thermofisher.com/antibody/product/Donkey-anti-Rat-IgG-H-L-Highly-Cross-Adsorbed-Secondary-Antibody-Polyclonal/A-21209)<br>Biotin-conjugated goat anti-mouse IgG antibody (BA-2000, Vector Laboratories), IHC (https://vectorlabs.com/biotinylated-horse-anti-mouse-igg-antibody.html)<br>Biotin-conjugated goat anti-rabbit IgG antibody (BA-1000, Vector Laboratories), IHC (https://vectorlabs.com/biotinylated-goat-anti-rabbit-igg-antibody.html)<br>HRP-conjugated rabbit anti-mouse antibodies (SC-358914, Santa Cruz Biotechnology), WB (https://www.scbt.com/p/rabbit-anti-mouse-igg-hrp?productCanUrl=rabbit-anti-mouse-igg-hrp)<br>HRP-conjugated goat anti-rabbit antibodies (SC-2054, Santa Cruz Biotechnology), WB (https://www.scbt.com/p/goat-anti-rabbit-igg-hrp?productCanUrl=goat-anti-rabbit-igg-hrp)

Animals and other organisms

Policy information about studies involving animals; ARRIVE guidelines recommended for reporting animal research

Laboratory animals	C57Bl/6 (WT) and GzmB-/- mice with C57Bl/6 background were obtained from Jackson Laboratory (Bar Harbor, ME, USA). GzmB-/- mice were maintained as homozygous colonies or by continuous backcross to C57Bl/6 mice.<br>7-10 week-old female and male mice were used for EBA studies.<br>Humanized COL17 mice (mCol17-/-, hCOL17Tg/Tg) were generated as described elsewhere and were maintained as homozygous colonies. WT, GzmB-/-, and humanized COL17 mice were bred and housed at the Genetic Engineered Models facility, St. Paul's Hospital, UBC or at ICORD, UBC.<br>1 day-old female and male mice were used for BP study.<br>All mice were housed in a 12 h light/dark cycle with controlled room temperature at 20-26C and 40-60% relative humidity.<br>All procedures were performed in accordance to the guidelines for animal experimentation approved by the Animal Experimentation Committee of UBC. Double transgenic Cre/iDTR mice were generated by crossing Mcpt5-Cre mice (C57Bl/6 background) with the iDTR line as described elsewhere. This strain was bred and housed at the animal facilities of the Research Center Borstel.
Wild animals	The study did not involve wild animals.
Field-collected samples	The study did not involve samples collected from the field.
Ethics oversight	All procedures were performed in accordance to the guidelines for animal experimentation approved by the Animal Experimentation Committee of UBC.

Note that full information on the approval of the study protocol must also be provided in the manuscript.

Human research participants

Policy information about studies involving human research participants

Population characteristics	We did not collect covariate-relevant population characteristics such as age, gender, genotypic information, past diagnosis, and treatment categories, except current diagnosis as pemphigoid disease, because we did not perform interventional studies and as this would not have modified our analyses. BP diagnosis was made based on clinical, histological, and immunopathological findings by dermatologists at the Dermatology Department in Vancouver General Hospital and Osaka City University Hospital.
Recruitment	For prospective collection, patients under care at Dermatology Departent in Vancouver General Hospital and Osaka City University Hospital were recruited by dermatologists. For retrospective collection, dermatologists at Dermatology Department in Vancouver General Hospital and Osaka City University Hospital collected stocked samples. We do not expect any bias during the recruitment as the biospecimens were analyzed by independent researchers.
Ethics oversight	In accordance to Vancouver General Hospital (Vancouver, BC, Canada) and Osaka City University Hospital (Osaka, Japan) research guidelines and policies, we obtained informed patient consent from each participant. All experimental procedures using human samples were approved by the University of British Columbia (UBC) Review Ethics Board and Osaka City University Hospital Review Ethics Board.

Note that full information on the approval of the study protocol must also be provided in the manuscript.
